# Do Human Endogenous Retroviruses Contribute to Multiple Sclerosis, and if So, How?

**DOI:** 10.1007/s12035-018-1255-x

**Published:** 2018-07-25

**Authors:** Gerwyn Morris, Michael Maes, Marianna Murdjeva, Basant K. Puri

**Affiliations:** 1 IMPACT Strategic Research Centre, School of Medicine, Barwon Health, Deakin University, Geelong, Victoria Australia; 2 Department of Psychiatry, Faculty of Medicine, Chulalongkorn University, Bangkok, Thailand; 3 Department of Microbiology and Immunology, Medical University, Plovdiv, Bulgaria; 4 Department of Medicine, Hammersmith Hospital, Imperial College London, London, UK

**Keywords:** Human endogenous retrovirus, Multiple sclerosis, Molecular neurobiology, Inflammation, Oxidative stress, Epigenetic regulation

## Abstract

The gammaretroviral human endogenous retrovirus (HERV) families MRSV/HERV-W and HERV-H (including the closely related HERV-Fc1) are associated with an increased risk of multiple sclerosis (MS). Complete HERV sequences betray their endogenous retroviral origin, with open reading frames in *gag*, *pro*, *pol* and *env* being flanked by two long terminal repeats containing promoter and enhancer sequences with the capacity to regulate HERV transactivation and the activity of host genes in spite of endogenous epigenetic repression mechanisms. HERV virions, RNA, cDNA, Gag and Env, and antibodies to HERV transcriptional products, have variously been found in the blood and/or brain and/or cerebrospinal fluid of MS patients, with the HERV expression level being associated with disease status. Furthermore, some HERV-associated single nucleotide polymorphisms (SNPs), such as rs662139 T/C in a 3-kb region of Xq22.3 containing a HERV-W *env* locus, and rs391745, upstream of the HERV-Fc1 locus on the X chromosome, are associated with MS susceptibility, while a negative association has been reported with SNPs in the tripartite motif-containing (TRIM) protein-encoding genes *TRIM5* and *TRIM22*. Factors affecting HERV transcription include immune activation and inflammation, since HERV promoter regions possess binding sites for related transcription factors; oxidative stress, with oxidation of guanine to 8-oxoguanine and conversion of cytosine to 5-hydroxymethylcytosine preventing binding of methyl groups transferred by DNA methyltransferases; oxidative stress also inhibits the activity of deacetylases, thereby favouring the acetylation of histone lysine residues favouring gene expression; interferon beta; natalizumab treatment; impaired epigenetic regulation; and the sex of patients.

## Introduction

Human endogenous retroviruses (HERVs), which are derived from previous exogenous retroviral infections, together with ERV-like DNA sequences make up about 8% of the human genome, distributed at approximately 700,000 different loci [1]. HERV classification has been a subject of considerable controversy [2] but the most recent analysis of HERV elements in the human genome indicates that HERVs consist of the following three main classes based on sequence similarity with their exogenous retrovirus counterparts, the fit with published clades and taxonomic markers: class I consists of *Gammaretrovirus*- and *Epsilonretrovirus*-like HERVs; class II consists of *Betaretrovirus*-like HERVs; and class III with one member HERV-L (HERV with leucine tRNA primer), which is distantly related to the *Spumaretrovirus* genus [3]. Each class encompasses a variable number of groups [3]. At the time of writing, bioinformatics-based approaches have identified 103 HERV families, although only 40 HERV families have been characterised in laboratory studies [4, 5].

**Fig. 1:**
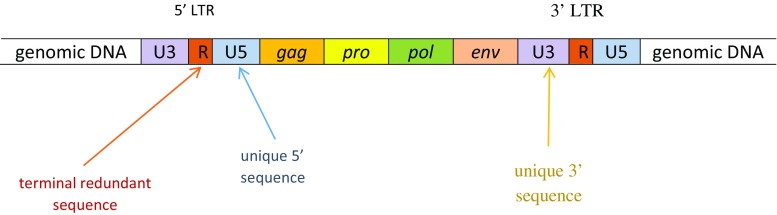
Diagrammatic depiction of typical HERV DNA sequences (not to scale)

Complete HERV sequences betray their endogenous retroviral origin, with open reading frames (ORFs) in *gag* (the group-specific antigen gene), *pro* (the protease gene), *pol* (the polymerase gene) and *env* (the envelope gene) being flanked by two long terminal repeats (LTRs) containing promoter and enhancer sequences with the capacity to regulate HERV transactivation and the activity of host genes [6] (see Fig. 1). However, over millions of years, integrated HERV sequences have accumulated mutations in their ORFs leaving them replication defective and for the most part unable to move within the genome. In addition, the original proviruses have undergone extreme recombination events often leaving the original virus represented by a solo LTR [7, 8].

Solo LTRs appear to have been selected for because of their positive role in the regulation of host genes [9]. Thousands of cellular transcripts initiated at HERV LTRs and these sequences are involved in the regulation of a myriad of genes [10, 11]. In particular, HERVs and their LTRs can provide promoters (alternative, sometimes bidirectional), enhancers, repressors, poly(A) signals and alternative splicing sites for human gene transcripts [12, 13]. HERV proviral sequences also modulate the activity of nearby genes and have the capacity to regulate the genomic regulatory landscape via a number of mechanisms such as providing transcription factor binding sites [14, 15].

However, while the retention of HERV sequences in the human genome is likely because the beneficial effects on the species outweigh any detrimental effects in individuals [5, 16, 17], the danger of inappropriate HERV expression to individuals may be considerable. For example, HERV expression can initiate and increase the activation of immune and inflammatory pathways [16, 18–20] and dysregulate gene pathways by affecting the levels of DNA transcription factors such as cAMP (cyclic adenosine monophosphate) response element-binding protein (CREB) and nuclear factor kappa-light-chain-enhancer of activated B cells (NF-κB) [21, 22]. Abnormal HERV expression can also potentially compromise neurotransmission and brain chemistry [23, 24].

Unsurprisingly, HERV expression is curtailed in healthy individuals by epigenetic machinery including GC methylation [25–27], histone modifications and RNA silencing [28–30]. However, despite this level of epigenetic repression, HERVs continue to be expressed in the periphery and in the brain [30–34]. Moreover, HERVs are transcribed at high levels in rheumatoid arthritis, Sjögren’s disease, systemic lupus erythematosus (SLE), schizophrenia and multiple sclerosis (MS), and are proposed to play a major role in the pathogenesis of these illnesses (reviews [30, 35–39]). HERV activity may impact on the diseases by the expression of RNA, cDNA, functional immunogenic proteins, superantigens and abnormal gene activation [9, 35].

## Disease Processes and Stimulation of HERV Expression

### HERV Transcription in MS

Two ‘Gammaretroviral’ HERV families have been predominantly related to MS, namely MRSV/HERV-W (murine retrovirus/HERV with tryptophan tRNA primer) and HERV-H (HERV with histidine tRNA primer), although the association of a close relative of HERV-H namely HERV-Fc1 and HERV-K-18 and the risk of MS has also been documented [20, 38, 40].

Several studies have revealed the presence of HERV-H virions [41, 42] and MSRV/HERV-W virions [43, 44] in MS patient blood samples. Elevated levels of HERV-H, HERV-K and HERV-W RNA have also been detected in MS patient brains [75]. HERV-W Gag and Env proteins have also been detected in MS patient brain tissue [45, 46].

The MSRV virion was the first member of the HERV-W family associated with MS and indeed the first member of the HERV-W family described [47]. The discovery of what initially appeared to be a new class of exogenous retrovirus provoked considerable debate and controversy regarding its origin which still persists at the present time [20, 48, 49]. For example, some authors have proposed that its origin could stem from the expression of isolated genes such as the one now known as *ERVW1*, which encodes the cell-cell fusion protein syncytin-1 [46], while other research teams have suggested the existence of extracellular MSRV particles [50] or even possibly novel exogenous retrovirus as previously discussed [48, 51]. The matter was apparently settled by evidence supplied by Laufer and colleagues, who supplied evidence which suggested that all listed MSRV *env* sequences originated at HERV-W *env* Xq22.3 [52]; known HERV-W *env* loci or recombinations among them most likely involving the HERV-W *env* locus are located on chromosome Xq22.3, which rather argued for the creation of the MSRV virion by in vitro recombination events during polymerase chain reaction (PCR) [52]. However, this conclusion has been challenged by recent work conducted by Grandi and fellow workers who have identified 16 full-length *env* sequences and a further 10 conserved but truncated *env* sequences in the human genome [53]. Moreover, these authors have adduced evidence suggesting that MRSV *env* could originate from as many as six different loci [53].

The same research team have reported the presence of 213 HERV-W elements in the human genome with 80 elements inserted into coding genes and 25 into noncoding regions [53]. HERV-W LTRs act as promoters in directing transcription of HERV-W members as is the case with other HERV proviral sequences [54]. However, in contrast to other HERVs, leaky transcription from adjacent genes plays a major role in the transcription of HERV-W proviral sequences lacking LTRs often described as pseudoelements or pseudogenes [54]. It is noteworthy that in contrast to all other known HERV groups, HERV-W pseudogenes and proviral transcripts have the unique capacity to be transposed by long interspersed nuclear element (LINE)-1 (L1) human retrotransposons [53, 55]. Given that the genome contains approximately 80–100 L1 elements still competent for retrotransposition [56–58], the potential for mobilising HERV-W elements in the genome leading to phenomena akin to insertional mutagenesis, or at least temporally variable patterns of gene regulation, would appear to be significant and a source of potential pathology which appears to be under-researched [59, 60].

### MSRV and HERV-W Expression in MS and Association with Disease Status

The proportion of MS patients testing positive for the presence of MRSV sequences in serum or plasma appears cohort-, and perhaps technique-, dependent, with ranges from 100% [61] to 50% [44] being reported. Enhanced levels of MSRV have been reported by researchers examining MS plaques post-mortem [45, 46, 62] and the presence of MSRV virions in the blood and cerebrospinal fluid (CSF) of MS patients has been reported by several groups [43, 61, 63–66]. The presence of MSRV has also been reported in patients with other neurological conditions, albeit at lower frequencies [21, 63, 65, 67], and in a small minority of disease-free volunteers [44, 63]. Dolei and fellow workers reported the presence of MRSV in the plasma in 100% of a patient cohort with active MS [63]. Moreover, the presence of MSRV particles in CSF paralleled progression of the illness with an increased level in relapse which fell to almost undetectable levels during remission [63, 68]. It also appears that patients without MSRV in the CSF tend to have stable disease whilst those with high CSF levels present with more severe disease patterns, multiple relapses and a poorer prognosis requiring multiple treatment when monitored over a 10-year period [66, 69, 70].

Further *prima facie* evidence of poor prognosis associated with the presence of MSRV in the CSF has been supplied by Sotgiu and fellow workers who reported that the presence of the virion in the CSF of monosymptomatic optic neuritis patients was strongly predictive of a conversion to definite MS [69]. It is also noteworthy that increased MSRV DNA copy number in MS patient blood samples is also associated with a poorer prognosis [71, 72]. Furthermore, interferon (INF) β-1 treatment reduces MSRV load in treatment responders and it has been proposed that the MRSV level could act as a biomarker for therapy outcome and disease progression [68, 73]. Garcia-Montojo and colleagues reported a sex-specific pattern of MRSV transcription. These authors predictably detected a higher load of MRSV in male and female patients with MS compared with healthy male and female controls [71]. However, the MRSV load was higher in female patients with active illness than males and, importantly, levels of MRSV correlated positively with Expanded Disability Status Scale (EDSS) and Multiple Sclerosis Severity Score (MSSS) ratings [74]. Notably, levels of MRSV/HERV-W expression were also higher in females than males in the control group [71].

Other HERVs are also present in activated form in vivo in MS patients. This is based on the demonstration of activated HERV-H virions in blood from MS patients [41, 42] and increased levels of HERV-H and HERV-K in MS brains [75]. HERV-W/MRSV is transcribed in B lymphocytes, monocytes and natural killer (NK) cells in patients with relapsing-remitting MS (RRMS), but not in T lymphocytes [76–79].

### Antibodies to HERV Transcriptional Products in MS

Brudek and colleagues were the first to report the skewed pattern of expression of MSRV/HERV-W in the peripheral blood mononuclear cells (PMBCs) of MS patients [79]. These authors reported a significantly greater expression of HERV-H and HERV-W Env epitopes on the surface of B cells and monocytes but not T cells from patients during relapse compared with both patients in remission and matched controls (both healthy controls and controls who suffered from a different neurological illness, namely epilepsy) [79]. Furthermore, MS patients in relapse had higher antibody reactivities directed towards HERV-W and HERV-H Env epitopes and a higher proportion of B cells in the PMBC fraction. Importantly, higher reactivities of the antibodies in sera from relapsed MS patients correlated positively with the higher levels of HERV-H Env and HERV-W Env expression on B cells and monocytes but such a correlation was absent for patients in remission [79]. Very similar findings were reported in a later study by the same team of researchers [80]. Increased CSF and serum levels of antibodies directed towards HERV-H Gag and Env have been reported in MS [81–83]. The presence of anti-MSRV/HERV-W antibodies in patient sera has also been reported [84].

### HERV-Associated Polymorphisms and MS Susceptibility

García-Montojo and fellow workers reported a positive association between the presence of the single nucleotide polymorphism (SNP) rs662139 T/C in a 3-kb region of Xq22.3 containing a HERV-W *env* locus and increased susceptibility to MS in females in a study involving 1669 MS patients and 1458 controls who were matched for age, sex and ethnicity [74]. The presence of this polymorphism in females was also associated with increased levels of MSRV and disease severity [74]. This polymorphism was associated with an increased susceptibility to MS and increased illness severity. Several other research teams have also reported a negative association between SNPs in the tripartite motif-containing (TRIM) protein-encoding genes *TRIM5* and *TRIM22* on chromosome 11 and MS susceptibility, and a positive association with the SNP rs391745, one of a cluster of SNPs in a region lying upstream of the HERV-Fc1 locus on the X chromosome [40, 85, 86]. It is noteworthy that no microRNA (miRNA) genes have been reported in this region and the nearest known genes lie 147 kb upstream and 57 kb downstream respectively [40]. When considered as a whole, this evidence is suggestive of HERV involvement in the pathogenesis of MS but is far from conclusive. Similarly, while the evidence associating HERV-W/MRSV expression with MS severity and/or risk appears robust, such expression could clearly be secondary to internal factors within MS patients and/or secondary to the disease processes driving the development and maintenance of the illness. A consideration of these factors forms the next topic of this discussion.

## Factors Affecting HERV Transcription

### Systemic Immune Activation and Inflammation

HERV promoter regions possess binding sites for a range of transcription factors involved in regulating the duration and intensity of the immune response. Such transcription factors include GATA factors (which bind the GATA DNA sequence) [87], activator protein (AP)-1 and AP-2 [88, 89], IFN regulatory factors (IRFs) 1, 2 and 7 [90], signal transducer and activator of transcription (STAT) factors [91], p53 isoforms [92], the nuclear factor of activated T cells (NFAT) family [93] and NF-κB [94, 95]. Importantly, these molecules are all major players in regulating and/or effecting the innate immune response and hence the expression of HERV elements is increased during acute or chronic immune activation.

### Oxidative Stress

Oxidative stress exerts a range of effects on chromatin and DNA methylation levels which broadly favour HERV expression (reviewed [96, 97]). One such mechanism which promotes DNA hypomethylation involves oxidation of guanine to 8-oxoguanine, and the conversion of cytosine to 5-hydroxymethylcytosine, thereby preventing the binding of methyl groups transferred by DNA methyltransferases (DNMTs) [98].

**Fig. 2:**
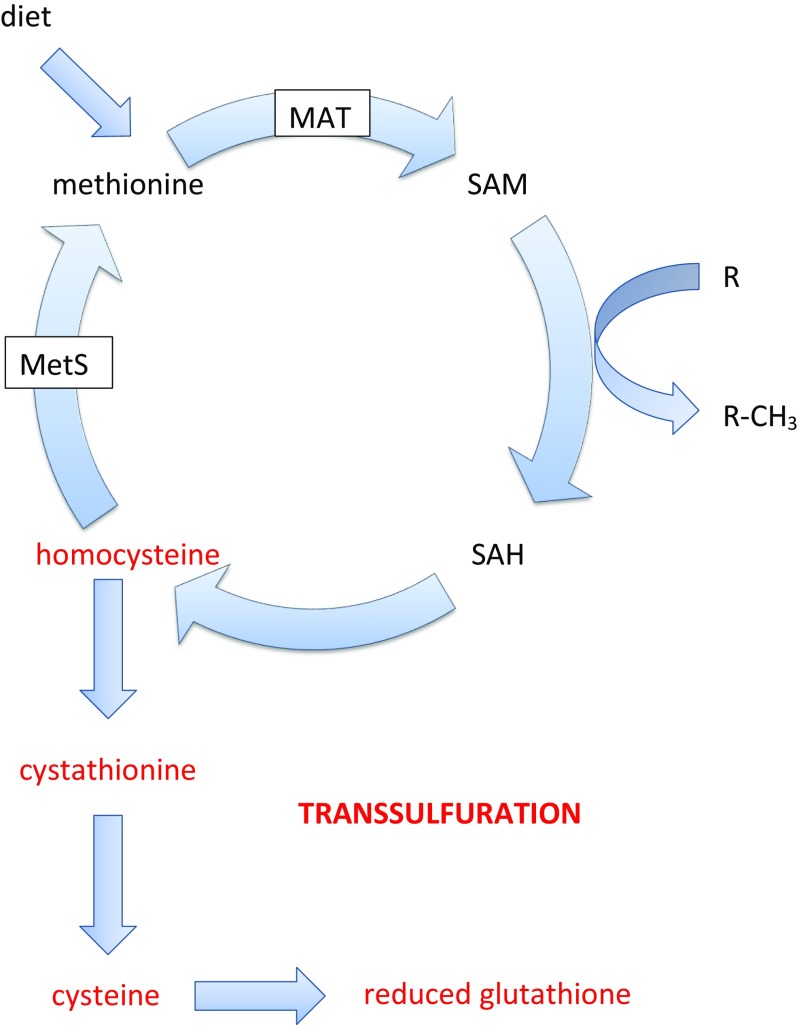
Aspects of the methionine and homocysteine metabolic cycle, showing the and transsulfuration pathway in red. SAH, *S*-adenosylhomocysteine; MetS, methionine synthase. Note that not all associated reactions are shown

DNMTs utilise *S*-adenosylmethionine (SAM) to transfer a methyl group to cytosine, and crucially, DNA methylation is directly inhibited when SAM levels and/or activity decrease [96]. Under physiological conditions, SAM is synthesised from methionine by the enzyme methionine adenosyltransferase (MAT). SAM is then utilised as a methyl group donor for methylation reactions, leading to the production of *S*-adenosylhomocysteine, which is then hydrolysed to homocysteine. The regeneration of methionine from homocysteine by methionine synthase completes the methylation or one-carbon cycle (reviewed [99]). Oxidative stress-induced depletion of SAM can be triggered by several mechanisms. First, oxidation of cysteine groups within the active site of MAT leads to its inactivation and thus to decreased SAM production from methionine [100, 101]. Second, the cobalamin group in methionine synthase, which acts as an essential cofactor, is readily oxidised, which unsurprisingly leads to inactivation of the enzyme [102]. Third, under conditions of oxidative stress, the transsulfuration pathway regenerates reduced glutathione from SAM via homocysteine, cystathionine and cysteine, thereby depleting SAM levels in the process [103–105]. Key aspects of these reactions are illustrated in Fig. 2.

Oxidative stress also inhibits the activity of class I, II and III deacetylases, thereby favouring the acetylation of histone lysine residues favouring gene expression [106–108]. Oxidative stress also decreases the catalytic activity of the JmjC family of histone lysine demethylases, once again favouring H3K4 methylation (that is, methylation of lysine 4 of the histone protein H3) and transcriptional activation [109, 110].

### CNS Immune Activation and Oxidative Stress

MS is characterised by the presence of activated microglia and astrocytes leading to the development of chronic inflammation. Neuroinflammation is maintained and exacerbated via the upregulation of proinflammatory cytokines (PICs), reactive oxygen species (ROS), tryptophan catabolites (TRYCATs), prostaglandins and prostaglandin-endoperoxide synthase 2 (PTGS2 or cyclooxygenase-2 (COX-2)) produced by activated glial cells [111–113]. Moreover, chronic neuroinflammation is also characterised by chronically upregulated transcription and activation of NF-κB, which is known to transactivate HERVs as discussed above [114–116].

B lymphocytes from MS patients display abnormal and exaggerated PIC responses, including an elevated ratio of lymphotoxin (LT) to interleukin (IL)-10, and enhanced secretion of LT and tumour necrosis factor alpha (TNFα) when activated by INF-γ or via toll-like receptor 9 (TLR9) engagement induced by the pathogen-associated molecular pattern (PAMP) ligand CpG-DNA [117]. B cells from MS patients also produce decreased amounts of IL-10 and excessive levels of PICs such as TNFα and IL-6, which could also account for elevated HERV transcription in these lymphocytes [118, 119]. The presence of activated B cells and follicular helper T cells together with elevated levels of IL-17 and PIC-secreting Th17 cells generate systemic inflammation, the levels of which correlate positively with disease severity and disease progression [120]. While activated glial cells are responsible for maintaining the chronic neuroinflammation seen in MS, the contribution of recently discovered follicle-like regions, containing follicular helper T cells, B cells and follicular dendritic cells, seen in the brains of some people with the illness, to this inflammatory state is being increasingly recognised [121]. Importantly, the presence and numbers of such centres correlate with the progression to severe disease, lower age of onset and level of central nervous system (CNS) inflammation [121]. When considered as a whole, elevated levels of systemic and CNS inflammation with the presence of elevated TNFα, IL-6 and NF-κB would account for higher levels of HERV activity in the brains of patients with MS than healthy controls.

Moreover, the higher levels of inflammation seen in people who progress to active disease and have a poor prognosis would explain the high levels of MRSV seen in such patients. In this case, the presence of MRSV in the CNS seen in such patients could well act as a biomarker for disease severity but not necessarily a driver of pathology. Chronic systemic inflammation invariably co-occurs with chronic oxidative stress [122] and this state can also influence the transactivation of HERVs, as previously discussed. Furthermore, chronic oxidative stress is also causatively implicated in the pathogenesis of MS [123] and we will now consider this phenomenon both from the perspective of disease process and increased transactivation of MRSV/HERV-W and HERV-H.

Levels of oxidative stress and cytokines correlate with levels of disability as measured by the EDSS [124]. Oxidative stress is present in the brains and PMBCs of patients with RRMS, and the extent of imbalance between the production of oxidative and nitrosative species and antioxidant defences is predictive of the course of the disease [123]. Crucially, levels of oxidative stress increase prior to and during relapse and markedly decrease prior to entry into remission [125]. It is also of relevance that PMBCs of female patients produce greater levels of ROS than those of male patients and display lower levels of membrane-bound antioxidants [123]. This is perhaps unsurprising given the role of oestrogen in upregulating the production of ROS by mitochondria [126, 127]. It seems reasonably clear that elevated levels of oxidative stress during relapse could account for data indicating upregulated transcription of HERV-W/HERV-H in patients during this phase of their disease and the correlation between MRSV levels and markers of disability. It is also plausible that elevated levels of oxidative stress during relapse may even account for increased activity in female over male patients in this phase of the illness. The immune and inflammatory imbalances seen in people with MS and their effect on HERV transcription also potentially explains the reduction in HERV expression in good responders to IFN-β therapy and natalizumab, as we will now discuss.

### Interferon Beta

IFN-β-1b suppresses levels of CD40 and CD80 expression on B cells in patients with RRMS and hence their stimulatory capacity. Moreover, IFN-β-1b inhibits B cell production of IL-1β and IL-23 but upregulates IL-12, IL-27 and IL-10. Its capacity to suppress the transcription of *RORC* (the gene for retinoic acid-related orphan nuclear hormone receptor C) likely underlies its ability to downregulate the production of IL-17 [128, 129]. INF-β therapy also downregulates the production of PICs by dendritic cells and upregulates the production of IL-12 and IL-23, hence shifting the cytokine environment which inhibits the differentiation of IL-17-producing Th17 T cells [128]. There appears to be a deficit in endogenous INF-β-regulated T cell homeostasis in some people with RRMS which is corrected by INF-β therapy via the upregulation of suppressor of cytokine signalling 3 (SOCS3) [130]. Given the major contribution of Th17 T cells to the upregulation of NF-κB via the production of TNFα, and 1L-1β, it is likely that inhibition of this pathway is a major reason for the reduction in HERV transcription seen after INF β therapy.

### Natalizumab

The apparent capacity of the monoclonal antibody natalizumab (NAT) to inhibit the transcription of HERV-W/MSRV *env* [131] may be the result of several effects of the drug, not least its capacity to modulate the transcription of miRNAs. For example, miRNAs which are downregulated in T cells during active disease are upregulated by NAT during remission [132]. Conversely, a number of miRNAs such as mR-126 and miR-17 are downregulated during NAT therapy upon entry into remission but are upregulated in patients during relapse [133, 134]. It is tempting to conclude that NAT therapy reduces the expression of HERV-W/MSRV in B cells and monocytes by changing the pattern of miRNA expression, and indeed, this may be part of the mechanism underpinning this phenomenon. The capacity of NAT therapy to inhibit the expression of HERV-W/MSRV in B cells, monocytes and NK cells in patients with RRMS without any changes in B cell numbers is also of interest [76].

However, NAT administration affects many other parameters known to be involved in HERV silencing or transactivation. For example, in MS patients, NAT therapy leads to the reduction of IL-1 and IL-8 in the CSF and a reduction of TNFα, IL-6 and a range of other proinflammatory molecules in serum [135, 136]. There is also copious evidence demonstrating that the therapeutic use of NAT inhibits the transcription and activation of NF-κB (reviewed in [137]). NAT administration also upregulates levels of haem oxygenase and nuclear factor erythroid 2-related factor 2 (Nrf2) thus enhancing cellular antioxidant systems and ameliorating oxidative damage to macromolecules and levels of oxidative stress [137]. Hence, NAT inhibits elements whose upregulation are known to transactivate HERVs, and downregulates oxidative stress; decreasing the expression of NF-κB and PICs likely underpins its capacity to silence HERV expression although a primary effect based on correcting abnormal miRNA expression cannot be ruled out.

### Impaired Epigenetic Regulation

An altered level and pattern of DNA methylation has been observed in the white matter of MS patients which is free of any obvious pathology [138]. Some genes involved in oligodendrocyte survival are downregulated and some involved in protein breakdown processing are upregulated [138]. DNA (cytosine-5)-methyltransferase 1 (DNMT1) activity is downregulated in PMBCs from patients with RRMS likely as a result of upregulated miR-21 [139–141]. The hypomethylated state of the *HLA-DRB1* locus in T cells of patients with RRMS is a fairly recent discovery [142]. Moreover, increased levels of the epigenetic regulator histone deacetylase 3 (HDAC3) occur in T cells of RRMS patients and sirtuin 1 (SIRT1) is decreased during relapses in patients suffering from the illness [143]. Hence, the mechanisms normally involved in the repression of HERV expression are attenuated in patients with MS and this attenuation appears to be greater during remission, potentially accounting for differentially increased expression of MRSV/HERV-W in this phase of the illness. This would certainly go some way to explaining the various increased manifestations of HERV activity during relapse compared with remission seen in several studies. Impairments in the molecular machinery governing the transcriptional downregulation of HERVs would explain the high levels of HERV-Fc1 expression [38] in the illness in particular as the role of *DNMT1* and the maintenance of methylation in repressing its expression has been established [38]. The relative increase in histone acetylation following the downregulation of *SIRT1* during relapse would go some way to accounting for the fourfold increase in expression of the provirus during relapse compared with stable disease as deacetylation of histone in the promoter region leads to a fourfold increase in proviral expression in cell lines [38]. It is also worthy of note that there are significantly reduced levels of global DNA methylation in females compared with males [144, 145] and this hypomethylated state could go some way to explaining why MRSV/ HERV-W is transcribed at a higher level in healthy females compared with healthy males. The sex of patients is known to play a role in the pathogenesis and pathophysiology of MS, and thus, we will now discuss the potential effects of this parameter on HERV activity in the illness.

### Sex Differences

The cellular and humoral immune responses following antigen challenge are more vigorous in healthy females compared with healthy males [146–148]. Sex biases in the immune system between males and females include higher numbers of B cells, higher levels of immunoglobulins and stronger antibody responses in females compared with males [149, 150]. This sex bias also extends to cytokine production, with basal levels of IL-1β, INF-γ and IL-4 being significantly higher in the female compared with the male population [151]. The pattern and levels of gene expression in resting immune cells are also strikingly different [150]. In particular, genes such as *TNFRSF17*, pivotally involved in the activation of NF-κB and Jak-STAT (Janus kinases/signal transducer and activator of transcription protein) signalling pathways, are significantly upregulated following stimulation in B cells extracted from females, which is a pattern not seen in B cells extracted from males and likely related to oestrogen levels [150]. Males have almost four times the number of circulating Foxp3 regulatory T cells than females [152, 153] and higher numbers of regulatory T cells per se [154]. Levels of IL-17A-positive Th17 T lymphocytes also appear to be higher in females compared with males in a Th17 polarising environment [155]. Finally there is evidence of increased levels of PICs in females compared with males in patients in RRMS and secondary progressive MS [156].

When viewed as a whole, it is likely that the level of inflammation as evidenced by PICs and NF-κB is higher in female than male MS patients and that this state of affairs may go some way to explaining higher levels of HERV-W/MRSV levels in female patients than in males.

There is now considerable evidence that a wide array of molecular abnormalities involved in the pathophysiology of RRMS differ between females and males. For example, several genetic factors confer an increased risk for the development of the illness in a sex-dependent trajectory [157]. Furthermore, a range of genes governing the performance of bioenergetics and inflammatory pathways are differentially expressed between males and females with MS, which have a mirror pattern of activation between relapse and remission [158]. Moreover, there is now evidence that Epstein-Barr virus (EBV) is reactivated in B cells of female RRMS patients during relapse but this phenomenon is not seen in the B lymphocytes of male patients [158].

This observation is important as EBV-encoded glycoprotein 350 (EBV-gp350) induces the expression of HERV-W/MSRV/syncytin-1 in astrocytes, apparently secondary to the upregulation of the NF-κB pathway. EBV-gp350 exposure leads to the transactivation of MRSV/HERV-W in B lymphocytes and monocytes but once again not in T cells nor indeed the normally highly expressing NK cells [68]. Levels of HERV-W/MRSV transcription are almost two orders of magnitude higher in patients with infectious mononucleosis compared with healthy controls, which provides further evidence of the capacity of EBV to activate these HERVs [78].

Several herpesviruses have the capacity to stimulate the transactivation of HERV-W [68, 78, 159, 160]. It appears that even inactivated herpesviruses have the ability to transactivate the expression of HERV virions in B cells and monocytes from MS patients, albeit in vitro [161]. Brudek and fellow workers reported elevated cellular immune responses towards a range of different HERVs and herpesvirus antigens concomitantly present in lymphocytes extracted from MS patients which were synergistic in nature [162]. Given this information and the data discussed above, it seems reasonable to conclude that HERV expression in MS could indeed result from sex effects, virus infections and the disease processes involved in MS. However, this does not exclude the possibility that MSRV/ HERV-W, HERV-Fc1 and HERV-H do play a role in the pathophysiology of the illness and we now move on to suggest mechanisms by which this could occur.

## Potential Contribution of HERV-W, HERV-H and HERV-Fc1 to MS Pathophysiology

### Virion and/or Protein Expression

Several research teams have reported the presence of MRSV/HERV-W virions with antigenic properties in at least some cultured cells extracted from MS patients [43, 47, 64, 163–165]. In addition, in vitro studies have established that MSRV/HERV-W Env and the MSRV virion can activate TLR4 on antigen presentation cells (APCs) including macrophages, dendritic cells and microglia leading to the production of a range of PICs such as IL-1β, TNFα and IL-6, which are known to play a causative role in the pathogenesis and pathophysiology of MS both in terms of microglial activation and actively provoking demyelination [166–169]. There are also in vitro data indicating that MSRV/HERV-W Env-mediated TLR4 stimulation also leads to the production of inducible nitric oxide synthase and the formation of nitrotyrosine residues secondary to high levels of peroxynitrite capable of inhibiting the differentiation of oligodendrocytes, thus suppressing myelin expression and renewal [170]. This latter finding has also been observed in brain tissue of MS patients and in mice with experimental autoimmune encephalomyelitis (EAE), in which HERV-W overexpression was associated with the development of neuroinflammation and damage to oligodendrocytes and myelin mediated by nitric oxide, peroxynitrite and other redox-active molecules [171, 172].

Injection of MRSV virions into genetically modified and immunosuppressed mice results in systemic immune activation and T cell-mediated neuropathology characterised by focal brain tissue destruction in parenchymal and meningeal tissue and, in many cases, haemorrhagic death [50]. These findings are supportive of earlier in vitro data demonstrating that MSRV virions and the Env protein can stimulate polyclonal T cell activation [173]. More recently, co-administration of MSRV-Env and myelin oligodendrocyte glycoprotein (MOG) in mice led to TLR4-mediated production of PICs and the development of EAE [174].

In addition, several authors have reported that HERV-W/MSRV Env displays properties of a superantigen capable of directly provoking T cell activation and proliferation leading to systemic immune activation and inflammation ([169, 173]; reviewed [175]). In the context of this finding, it is noteworthy that peripheral T cell activation and proliferation are known to play a major role in the pathogenesis of MS (reviewed [112]). It should be noted at this juncture however that while the data reviewed above are of interest, TLR4 activation in vivo would require the presence of extracellular MRSV virions or at least the Env protein, and the existence of MRSV in vivo is still a matter of considerable debate [52].

Expression of HERV-W Env protein has been repeatedly detected on the surface of microglia and macrophages in the brains of MS patients near or in actively demyelinating lesions [45, 172, 176]. Moreover, several regions of the Env protein show sequence homology with MOG and at least some of these sequences function as epitopes capable of provoking T and B cell activation [177, 178]. This is of importance as interactions between macrophages and microglia acting as APCs and autoreactive Th1 and Th17 T cells entering the CNS from the periphery are now considered to lie at the core of neuroinflammatory processes and myelin destruction in MS [179] (reviewed [113]).

Briefly, current data suggest that MS is driven by both Th1 and Th17 subsets, although each is mechanistically different from the other [9, 13]. Once activated, T cells traffic to the brain and cross the blood-brain barrier. In the brain, they are re-stimulated by APCs [14], leading to disease induction and progression. As disease progresses, new myelin antigens are presented by APCs (epitope spreading), leading to subsequent activation of newly infiltrated T cells [15]. These interactions include antigen presentation by microglia to activate T cells, the T cell activation of microglia, their progressive stimulation of one another, and the production of injurious or neurotrophic outcomes in their vicinity [180].

### RNA and/or cDNA Expression

**Fig. 3:**
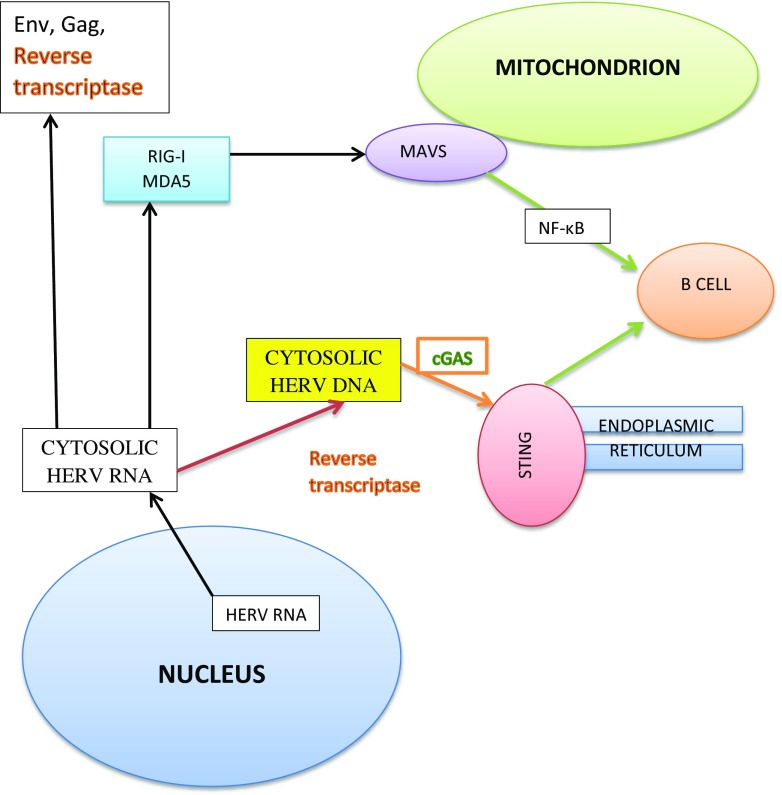
Some aspects of the host response to cytosolic HERV RNA and DNA

There is a considerable body of evidence demonstrating the presence of HERV-W/MSRV RNA and cDNA in samples taken from MS patients [52, 68, 71, 78, 181]. These findings are of importance given the presence of in vivo data demonstrating that HERV-W RNA can activate TLR3 on the endosomal membrane and melanoma differentiation–associated protein 5 (MDA5), protein kinase RNA-activated (PKR or eIF2AK2) and retinoic acid-inducible gene I (RIG-I) in the cytoplasm leading to the activation of NF-κB and the production of PICs and IRF3 and the production of type 1 interferons [182–184]. RIG-I and MDA5 are members of the RIG-I-like receptor (RLR) family, which recognise and activate in response to the presence of viral RNA in the cytoplasm. Those interested in more details of these receptors and their role in the immune response are invited to consult the work of [185]. From the perspective of this paper, however, the major point is that RIG-I recognises short dsRNA as well as ssRNA with a 5′-triphosphate group, whereas MDA5 detects and responds to long dsRNA molecules [186]. Some aspects of the host response to cytosolic HERV RNA and DNA are shown in Fig. 3.

A number of cytosolic receptors for DNA such as the DNA-dependent activator of IRFs (DAI) responsible for detecting long dsDNA, the absent in melanoma 2 (AIM2)-like receptors which detect dsDNA and IFN-γ-inducible protein 16 (IFI16) which bears responsibility for detecting ssDNA and subsequent activation of the adaptor stimulator of IFN genes (STING) leading to IFN-β production, have also been discovered and characterised [187–189]. Interestingly, IFI16 appears to be an important receptor responsible for detecting cDNA produced by reverse transcriptase during lentivirus replication [190]. Recent research has revealed the existence of other cytosolic DNA sensors such as DExD/H-box helicase 41 (DDX41) and cyclic GMP-AMP synthase (cGAS), with the latter also playing a major role in the detection of retroviral DNA and STING activation [191, 192]. However, there is no direct evidence that gammaretroviral HERV cDNA activates any of the cytosolic DNA sensors described above. However, the negative association between SNPs in both tripartite interaction motif 22 (TRIM22) and TRIM5 and the risk of developing MS could plausibly stem from altered regulation of RIG-I and mitochondrial antiviral signalling-5 (MAVS-5), or other cytosolic pattern recognition receptors in response to HERV RNA, as we now move to discuss.

## TRIM Family of E3 Ligases and Regulation of the Immune Response to RNA Viruses

The majority of the TRIM superfamily of E3 ligases play a major role in mediating signal transduction during the innate immune response to infection by viruses and other pathogens and the consequent production of pro- and anti-inflammatory cytokines ([193]; reviewed by [194]). Most TRIM proteins also have the ability to induce the transcription factors NF-κB and/or activator protein 1 (AP-1) [195]. Some TRIM proteins also play a regulatory role in the RNA helicase RIG-I-mediated interferon pathway lying upstream of the mitochondrial protein MAVS [195]. RIG-I, and other RNA helicases such as MDA5, detect cytosolic pathogen-associated RNA species and signal via MAVS to induce the phosphorylation and subsequent activation of IRF3 and IRF7, which act as transcription factors which stimulate the expression of type I interferon [196]. The activation of cytosolic RIG and nucleotide-binding oligomerisation domain (NOD)-like receptors also triggers the activation of NF-κB and PIC production via well-documented mechanisms [196]. *TRIM5* and *TRIM22* are closely associated on chromosome 11 and play somewhat different roles in regulating the immune response to retroviruses and a range of other RNA viruses [197].

TRIM5α, the α isoform of TRIM5, is an example of a TRIM functioning as both a direct virus restriction factor as well as a pathogen-recognition receptor [198]. TRIM5 activates transforming growth factor beta (TGF-β)-activated kinase 1 (TAK1), a downstream kinase utilised by RLRs to induce mitogen-activated protein (MAP) kinase and NF-κB signalling [195, 199, 200]. It seems that the capacity of TRIM5 to activate the innate immune system depends on the recognition of at least some exogenous or endogenous retroviral capsid amino acids but there would appear to be no requirement for the presence of a complete capsid [201]. This is of importance as HERV-Fc1 has an almost complete *gag* ORF and hence is likely to produce at least some capsid subunits [38]. This is particularly intriguing given that SNPs in the *TRIM5* locus within intron 1 have been found to be inversely associated with MS; intron 1 is immediately adjacent to the exon coding for a RING (really interesting new gene) finger domain [40]. There is a considerable body of evidence demonstrating the influence of SNPs on TRIM5 activity [202]; hence, the SNPs in question could plausibly reduce the strength of the immune response to HERV RNA and explain the reduction in risk of MS development associated with these polymorphisms.

*TRIM22* inhibits HIV-1 LTR-driven transcription via several mechanisms including the inhibition of binding of the transcription factor specificity protein 1 (Sp1) to promoter sequences [195, 203]. TRIM22 also regulates (NOD2)-dependent activation of IFN-β signalling and NF-κB upregulation [204]. The effects of TRIM22 in regulating the activation of the innate immune system appear to be pleiotropic however. For example, there is evidence suggesting that TRIM22 overexpression significantly upregulates NF-κB activity and the subsequent secretion of PICs by macrophages following TLR activation [205]. On the other hand, experimental evidence also suggests that TRIM22 negatively regulates TNF receptor-associated factor 6 **(**TRAF6)-stimulated NF-κB by inducing the degrading of TGF-β activated kinase 1 binding protein 2 (TAB2) [206]. This is of particular interest as experimental evidence suggests that TRAF6 is responsible for activating the innate immune responses via the activation of NF-κB and IRF7 upon sensing the presence of cytosolic viral RNA [207]. It is also worthy that this effect is inhibited by deletions in the RING domain [206]. Hence, polymorphisms in this TRIM family protein leading to overexpression could reduce NF-κB levels and thereby reduce inflammation and oxidative stress which might relate in part to reduced transcription of HERV-Fc-1 or MSRV/ HERV-W Env.

While the negative relationship between polymorphisms in *TRIM5* and *TRIM22* and the risk of developing MS could conceivably be related to altered immune responses in response to HERV transcription or even reduced HERV transcription, the positive relationship between MS risk and the polymorphism rs391745 upstream of the *HERV-Fc1* locus on the X chromosome [40, 85, 86] could also be connected to altered HERV expression, as we will now consider.

## Effects of rs391745 and Other SNPs on DNA Methylation and HERV Expression

The underlying genetic sequence affects DNA methylation and the presence of a CpG dinucleotide at a SNP influences the local pattern of DNA methylation [208] resulting in large disturbances in levels of DNA methylation near the polymorphism which is often described as a cis effect [208, 209]. The occurrence of SNPs can also lead to the creation of alternative polyadenylation signals ultimately affecting the expression of genes via a loss of miRNA regulation [210, 211]. Experimental evidence indicates that some 64% of transcribed SNPs have the capacity to increase or decrease the binding affinity of miRNAs by over 90% [212]. SNPs are also associated with more modest differences in the levels of DNA methylation at remote distances, demonstrating some evidence of trans effects [144]. There is also now considerable evidence that intragenic sequences not close to known genes may actually control transcription of unrecognised RNAs or distal genes [213].

There is widespread transcription across non-protein-coding regions in the human genome (for reviews, see [214, 215]). Such noncoding transcripts originate from introns, exons and intergenic DNA [216, 217]. The weight of evidence suggests that a great deal of this transcriptional activity plays a major role in regulating the transcription of distal genes [218]. It is now recognised that changes in the expression of DNA in intergenic regions may both activate or repress the transcription of geographically remote genes [219]. These noncoding regions of DNA can also regulate the expression of nearby genes and are often described as *cis*-regulatory elements (CREs) [220]. Such CREs can not only influence the expression of adjacent genes but also genes which are several hundred kilobases away [220]. Many polymorphisms in regulatory noncoding DNA sequences also regulate the expression of distal genes, which may even be on other chromosomes, and exert influences on both alleles of a given gene [220, 221].

L1 insertions also play a major role in gene regulation and are present at an extraordinary high level in the X chromosome and appear to play an important role in X chromosome inactivation [222, 223]. The activity of L1 is also under epigenetic control and “active” demethylated L1 insertions have the capacity to regulate the expression of proximal and distal genes at the transcriptional or post-transcriptional level [224]. Therefore, polymorphisms in L1 sequences could affect their activity and hence the activity of nearby or distal genes.

Given the data discussed above, the simplest explanation for the positive relationship between the presence of rs391745 upstream of the *HERV-Fc1* locus and increased MS risk could result from increased transcription of the provirus despite the remoteness of the SNP in question. However, there are other possibilities.

Genome-wide association studies have revealed a broad spectrum of non-HLA polymorphisms involved in increasing disease susceptibility (reviewed by [225]). For example, SNPs in or around loci containing the *IL2RA* and *IL7RA* genes are associated with increased risk of developing MS in French and German populations [226]. SNPs in *SOCS1*, *IL2RA*, *CD58*, *CLEC16A* and *FOXP3* are also independent predictors of MS susceptibility [226–228]. Given that *FOXP3* is located on the X chromosome, the association of a SNP in a region upstream from the *HERV-Fc1* locus with an increased risk of MS could result from decreased Foxp3 production.

Finally, sequence variation in the LTR or proviral region of HERV-W is known to affect its transcription [229, 230]. However, it is not at all clear that polymorphism rs662139 T/C in a 3-kb region of Xq22.3 (see above) occurs within the LTR or proviral regions of the HERV-W sequence found at this location. Nevertheless, given evidence of the remote influences of SNPs on transcription, it seems reasonable to conclude that this polymorphism leads to increased levels of MRSV *env* transcription, and in turn that this contributes to increased symptom severity, likely via TLR4 engagement and subsequent increases in inflammation and oxidative stress. The problem with this analysis is that it fails to account for the fact that this polymorphism is associated with an increased MS risk in females. Hence the possibility that this SNP is in a regulatory region and adversely influences methylation patterns associated with X chromosome inactivation, in some way accounting for higher transcription and MS risk limited to females. In addition, while this SNP could be responsible for increasing MRSV transcription, the increase in MS risk associated with the presence of rs662139 T/C could be secondary to an increase in inflammation, oxidative stress and localised DNA demethylation secondary to changes in activity in immune function genes, miRNAS or L1 sequences involved in the pathogenesis and/or pathophysiology of MS.

## Conclusion

In spite of endogenous epigenetic repression, HERVs are expressed in the human CNS. In particular, MRSV/HERV-W, HERV-Fc1 and HERV-K-18 are associated with an increased risk of MS; there is evidence of increased levels, in MS patients, of corresponding virions, RNA, cDNA and Gag and Env proteins. Indeed, the level of HERV expression is related to disease status, while some HERV-associated SNPs are associated with MS susceptibility. Since immune activation, inflammation and oxidative stress influence HERV transcription, measures to modulate these factors may offer further novel or adjunctive therapeutic options.

### Authorships

All authors contributed to the writing up of the paper.
